# Adherence to Mediterranean Diet and Its Association with Maternal and Newborn Outcomes

**DOI:** 10.3390/ijerph19148497

**Published:** 2022-07-12

**Authors:** Laura Di Renzo, Marco Marchetti, Giuseppe Rizzo, Paola Gualtieri, Diego Monsignore, Francesca Dominici, Ilenia Mappa, Ottavia Cavicchioni, Lorenzo Aguzzoli, Antonino De Lorenzo, on behalf of the MeDAP Group

**Affiliations:** 1 Section of Clinical Nutrition and Nutrigenomics, Department of Biomedicine and Prevention, University of Tor Vergata, Via Montpellier 1, 00133 Rome, Italy; laura.di.renzo@uniroma2.it (L.D.R.); diego.monsignore@gmail.com (D.M.); francescadominici@live.it (F.D.); delorenzo@uniroma2.it (A.D.L.); 2 PhD School of Applied Medical-Surgical Sciences, University of Rome Tor Vergata, Via Montpellier 1, 00133 Rome, Italy; marco@marcomarchetti.it; 3 Division of Gynecology and Obstetrics, Department of Biomedicine and Prevention, Tor Vergata University, 00133 Rome, Italy; giuseppe.rizzo@uniroma2.it (G.R.); mappa.ile@gmail.com (I.M.); 4 Fondazione Policlinico Tor Vergata, 00133 Rome, Italy; 5 Division of Maternal Fetal Medicine, Ospedale Cristo Re, Tor Vergata University, 00167 Rome, Italy; 6 Unit of Obstetrics and Gynecology, Ospedale S. Maria Nuova, 42123 Reggio Emilia, Italy; ottavia.cavicchioni@gmail.com (O.C.); aguzzoli.lorenzo@ausl.re.it (L.A.)

**Keywords:** pregnancy, obstetric outcome, neonatal outcome, birth weight, obesity, eating habits, lifestyle, Mediterranean diet

## Abstract

Background: Pregnancy is a crucial stage in a woman’s life and can be affected by epigenetic and environmental factors. Diet also plays a key role in gestation. This study aimed to evaluate how a greater or lesser adherence to the Mediterranean Diet (MD) influences specific parameters of mother and newborn. Methods: After delivery, the women participating in the study answered a questionnaire: demographic information; anthropometric data (pre-pregnancy weight, height, and gestational weight gain); dietary habits information (adherence to MD before and during pregnancy, using the validated Mediterranean Diet Adherence Screener (MEDAS), quality of protein intake); pregnancy information (onset of complications, cesarean/vaginal delivery, gestational age at birth, birth weight, birth length); and clinical practitioner for personalized dietary patterns during pregnancy. Results: A total of 501 respondents have been included in the study, and 135 were excluded for complications. Women who followed the advice of clinical nutritionists showed better adherence to MD (*p* = 0.02), and the baby’s birth weight was higher (*p* = 0.02). Significant differences in gestational weight gain (*p* < 0.01) between groups with dissimilar diet adherence were demonstrated. Conclusion: Our data demonstrate a significant relationship between adherence to MD and birthweight.

## 1. Introduction

Mounting evidence suggests that nutrition and lifestyle before and during pregnancy, breastfeeding, and childhood are responsible for long-term consequences on the child and adult health. These effects include a higher risk of developing non-communicable diseases, such as obesity, diabetes, and cardiovascular diseases (CVDs) [1]. This phenomenon is also known as “fetal programming”. It represents all endocrine-metabolic modifications in organs and tissues that arise during intrauterine life, and that could cause chronic-degenerative diseases (CDDs) [2,3,4,5]. The most accredited hypothesis is that the development of diseases in adult life could be linked to the type of feeding in the first stages of life, from intrauterine to organogenesis [6].

In the 1980s, Barker et al. described a link between the intrauterine environment and the risk of developing diseases in adult life. According to Barker’s predictive-adaptive model, the fetus programs its structure, organ function, and metabolism on environmental information received during intrauterine life, including maternal nutrition. It has been demonstrated that nutritional deficiencies during pregnancy, usually defined by insufficient intake of macronutrients or micronutrients, are related to abnormalities in fetal growth, CVD, hypertension, and type 2 diabetes (T2DM) [7,8].

Excessive gestational weight gain (GWG) is associated with several adverse perinatal outcomes, including abnormal fetal growth, risk of later childhood obesity, and a higher prevalence of preterm delivery, cesarean section (CS), gestational diabetes mellitus (GDM), hypertensive disorders of pregnancy and metabolic health outcomes [9,10]. Multiple randomized controlled trials have tested the efficacy of lifestyle intervention on GWG [11]. Despite lifestyle interventions that seem to alter weight gain, they have not been yet associated with improvement in perinatal outcomes [12].

The Mediterranean Diet (MD) is positively related with neonatal outcomes [13,14,15]. It has been demonstrated that MD is crucial and helpful for a correct gestational period and normal fetal growth. De Giuseppe et al. [16] have shown that high adherence to a MD is positively related to a lower incidence of Small for Gestational Age (SGA), which is improved by increased physical activity (PA) and changing some lifestyle habits (e.g., quitting smoking and alcohol consumption).

MD adherence has a protective effect on preterm delivery, better glucose regulation, and a good amount of folate, which is fundamental for neural tube development. Higher adherence to the MD during pregnancy is a potentially protective factor against abdominal obesity and positively influences lipoprotein and homocysteine concentration and insulin resistance in newborns [17].

Generally, for the women population, compliance with MD in association with PA is helpful to reduce the incidence of several diseases, such as obesity and CVDs, through Mediterranean personalized nutrition [18]. Therefore, knowing the same plausible starting mechanisms for one of the common pregnancy-related diseases, several studies have been focused on the association of diet and PA in the risk of developing GDM [19].

Moreover, MD could be considered a preventive and therapeutic medical protocol and a sustainable model related to country-specific and cultural variables for better health and nutritional benefits, environmental impact, and positive economic return [20]. The MD is an example of an accessible and sustainable diet. The nutritional program is low-cost and socially acceptable globally [21].

Based on this evidence, we started the Mediterranean Diet Adherence during Pregnancy (MeDAP) project with an online survey for women who gave birth in 2021.

The primary outcome of this study was to show MD effects on GWG and obstetrics and neonatal outcomes such as birth weight, birth length, and gestational age birth. The secondary outcome was to compare MD adherence levels between women who have had clinical nutritionists’ follow-ups and women without nutritional guidelines.

## 2. Materials and Methods

### 2.1. Study Design and Participants

The Clinical Nutrition and Nutrigenomics Section carried out the MeDAP project, with the participation of the Department of Biomedicine and Prevention of the University of Rome Tor Vergata, the Division of Maternal Fetal Medicine of the Hospital Cristo Re Roma and the Unit of Obstetrics and Gynecology of Ospedale S. Maria Nuova Reggio Emilia, using an anonymous online semi-structured questionnaire developed using google forms (available online: https://docs.google.com/forms (accessed on 2 April 2021), Google, Mountain View, CA, USA) aimed to obtain data on eating habits and lifestyle during pregnancy.

Random sampling was used for the sample size, considering 0.1% of births, calculated based on the previous year.

An a priori power analysis was conducted using G*Power version 3.1.9.7 to determine the minimum sample size required to test the study hypothesis. Results indicated the required sample size to achieve 80% power for detecting a medium effect, at a significance criterion of α = 0.05, was N = 172 for *t* tests—Means: Difference between two independent means (two groups). Thus, the obtained sample size of N = 172 is adequate to test the study hypothesis.

The link to advertise the questionnaire was shared via e-mail and social media. The exclusion criteria were twin pregnancy, women affected by pregnancy complications, and vegetarian and vegan eating habits. A specific questionnaire on vegetable protein intake and a dedicated study should be required for vegetarian/vegan dietary patterns. All of the women who answered our questionnaire must necessarily have already given birth within one year at the two referral hospitals. Participants reported their diet in two different surveys, one before and one during pregnancy.

A total of 501 women answered the questionnaire. In compliance with the inclusion and exclusion criteria, 135 women’s questionnaires were excluded from statistical analysis due to pregnancy complications.

The study was conducted following national and international regulations and the Declaration of Helsinki (2000). All participants were informed about the study objectives and consented to process data on the privacy policy. Participants completed the questionnaire directly on the Google platform. To maintain and protect the confidentiality of the participants, the anonymous nature of the web survey does not allow any way to trace sensitive personal data, according to EU Regulations (General Data Protection Regulation, GDPR, 679/2016). According to Italian national regulations, the present web survey study did not require approval by the Ethics Committee.

### 2.2. Questionnaire

The MeDAP questionnaire included 79 self-reported questions divided into 6 sections: (1) personal data (age, nationality, educational qualification); (2) anthropometric information obtained fasting with light clothes (height, current weight, weight before pregnancy, gestational weight gain); (3) obstetric history (other pregnancies, spontaneous and voluntary interruptions of pregnancy, any pathologies occurred during pregnancy, spontaneous or cesarean birth, gestational age at birth, newborn hospitalization types, use of supplements during pregnancy); (4) medical history and drug therapy; (5) lifestyle habits during pregnancy (smoking, alcohol and drug use); (6) eating habits: (6a) diet adherence and related changes before and during pregnancy; (6b) adherence to the MD before and during pregnancy, using a validated questionnaire; (6c) weekly frequencies of animal and plant protein sources’ intake by 20 questions. Due to the lack of a specific questionnaire designed for pregnancies, we opted to use the one validated for the general population [22]. Once completed, each questionnaire was automatically uploaded to the Google platform, and, at the end of the study, the final database was downloaded as a Microsoft Excel sheet.

### 2.3. Validated 14-Item Questionnaire of Mediterranean Diet Adherence (MEDAS)

The MD’s adherence evaluation was assessed using the validated Mediterranean Diet Adherence Screener (MEDAS) [22]. This questionnaire consists of 14 questions related to food intake habits and frequency of consumption of foods that are typical and non-typical of the MD. Responses favorable to the MD adoption were scored as 1 point, while unfavorable responses were scored as 0. Based on the MEDAS score, participants were divided into three groups: low adherence (score 0–5), medium adherence (score 6–9), and high adherence (score ≥10) to the MD. Then we evaluated the improved, worsened, or unchanged adherence to the MD before and during pregnancy through a delta score, and participants were divided into two groups: improved or unchanged MD adherence (delta score equal to 0 or >1) and worsened MD adherence (delta score <1). Women were grouped based on their answers to the questionnaire among those who received or did not receive nutritional guidelines.

The questionnaire is reported in Supplementary Materials File S1.

### 2.4. Statistical Analysis

Data are represented as numbers and percentages for categorical variables. At the same time, the median and interquartile range is expressed in square brackets [IQR] or mean and standard deviation for continuous variables according to their distribution. The Shapiro–Wilk test was performed to evaluate the variable distribution. The Pearson correlation coefficient was calculated to assess the correlation between continuous variables. Bartlett’s or Levene’s tests were used to test the variances’ homogeneity.

The paired Wilcoxon signed-rank test assessed differences in MEDAS score between pre- and during pregnancy. Subsequently, the *t*-test for related samples or the Wilcoxon signed-rank test were used to determine the differences in the variables examined between the different groups. The graphic representation for qualitative variables was also described as frequencies (%) regarding an ideal situation (100% compliance) summarized in the radar plot graph. The statistical differences were evaluated by Pearson and McNemar Chi-square tests.

Instead, the Kruskal–Wallis rank-sum test was performed to compare continuous variables among two or more groups. For post hoc analyses, multiple pairwise comparisons were performed using Dunn’s test with Bonferroni correction.

Finally, a general linear model (GLM) was conducted to investigate the association and the future prediction between categorical variables (dependent) and continuous or categorical ones (independent).

A two-sided *p*-value of <0.05 was considered statistically significant. Statistical analysis was performed using R (CRAN, Rcmdr package, vers. 2.7-1) [23].

## 3. Results

The survey was conducted from May 14 to June 21, 2021, among women who gave birth within one year at the two referral hospitals. A total of 503 completed the questionnaire, and, after data validation, 501 answers were included in the study. No women were vegetarian or vegan. Two questionnaires were excluded because of twin pregnancy. Maternal age ranged between 19 and 53 years, of which 273 were <35 years (54.5%) and 228 were ≥35 years (45.5%). Complications of pregnancies occurred in 135 (26.9%) women. General characteristics and anthropometrics of mothers are reported in Table 1.

Nationality was predominantly Italian (95.1%), and only 4.99% were foreigners.

SGA was 8.8%, appropriate-for-gestational age (AGA) was 76.8%, and LGA was 14.4%. Admission to the neonatal intensive care unit (NICU) occurred in 27 (5.4%) newborns and to the special care neonatal unit (SCNU) in 11 (2.2%) newborns (Table 2).

No relationship was found between these variables and maternal age.

**Figure 1:**
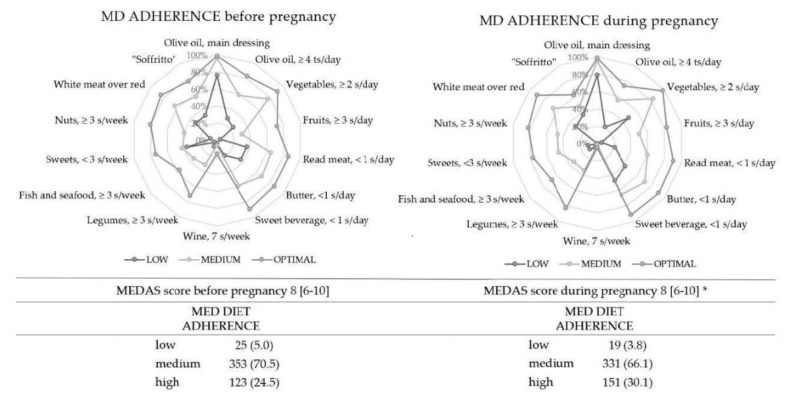
MEDAS items compliance following high, medium, and low adherence to MD before and during pregnancy. The radar graphic highlights every item value along the axis that starts in the center of the graph (compliance = 100%). Values are expressed as the percentage of the population that follows every recommendation. Vegetables daily serving: 1 medium portion = 200 g. Fruit daily serving: 1 serving = 100–150 g portion. Red meat/hamburgers/other meat daily serving: 1 medium portion = 100–150 g. Butter, margarine, or cream daily serving: 1 medium portion = 12 g. Sweet or sweetened carbonated drinks daily serving: 1 medium portion = 200 mL. Wine daily serving: 1 medium portion = 125 mL. Legumes weekly serving: 1 portion = 150 g. Fish daily serving: 1 medium portion = 100–150 g. Seafood daily serving: 1 medium portion = 200 g. Nuts weekly serving: 1 portion of dairy product = 30 g. MD, Mediterranean diet; s, serving; ts, tablespoon. * The Shapiro–Wilk test was performed to evaluate the variable distribution. Variables are considered non-normally distributed for *p* < 0.05.

The MEDAS questionnaire was used to evaluate the MD compliance recommendations before and during pregnancy. After sample partitioning into three groups (low, medium, and high adherence to MD), compliance differences were calculated for all foods, and they are represented illustrating the gap between sample answers and an ideal situation (100% of compliance) (Figure 1). MD adherence before pregnancy was 5.0%—low, 70.5%—medium, and 24.5%—high adherence in the study population; it was 3.8%—low, 66.1%—medium, and 30.1%—high adherence during pregnancy. There was an improvement in all parts. The McNemar test showed that women consumed more vegetables, fruits, nuts, and legumes (*p* < 0.001). No MD foods had been consumed less. The Wilcoxon-related *t*-test showed a higher MEDAS score during pregnancy with respect to before it (*p* < 0.001) (Figure 1).

**Figure 2:**
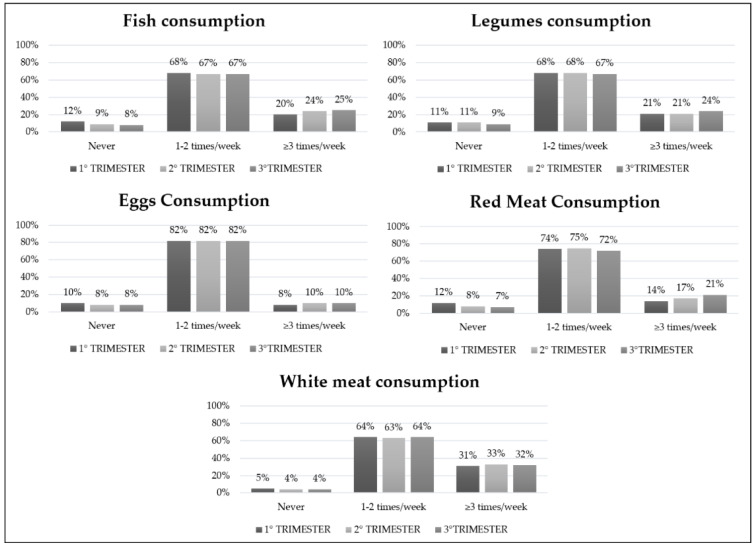
Animal and vegetable frequency intake during all pregnancy trimesters.

Analyzing the frequencies with the Pearson Chi-square test, a higher intake of white meat and red meat (respectively *p* < 0.001 and *p* = 0.02) was found in the second and third trimester. No statistically significant differences were observed in fish, eggs, and legumes consumption within the first, second, and third trimester (Figure 2).

We have also analyzed the MEDAS score and MD adherence of women grouped according to the source of nutritional information received by a clinical nutritionist (13.4%), gynecologist (15%), or no one (71.6%). The Kruskal–Wallis test showed a significant difference between groups (*p* = 0.02), with a higher score for women followed by clinical nutritionists (Bonferroni post-hoc, *p* = 0.03 and *p* = 0.04, respectively).

Statistical analysis of the pregnant women’s whole sample showed no significant changes in the birthweight and GWG. Nevertheless, a significant increase in the CS in the worsened MD adherence women was observed (*p* = 0.03). Therefore, the analysis for neonatal outcomes was restricted to uncomplicated pregnancies. When the delta score between MEDAS obtained before and during pregnancy was analyzed, an increased MD adherence was found in 270 (79.2%), while 76 (20.8%) showed a worsened adherence. The Chi-square test proved a significant difference between these two groups (*p* = 0.02).

Table 3 compares obstetrics and neonatal outcomes according to changes in MD adherence during pregnancy. Birthweight resulted differently among the two groups (*p* = 0.02), while no other differences were evidenced for other variables considered.

In the uncomplicated group, low adherence women (3.3%) had an average GWG of 16.4 kg, against an average of 12.8 kg for medium adherence (64.7%) and 11.9 kg for the high adherence group (32.0%).

**Figure 3:**
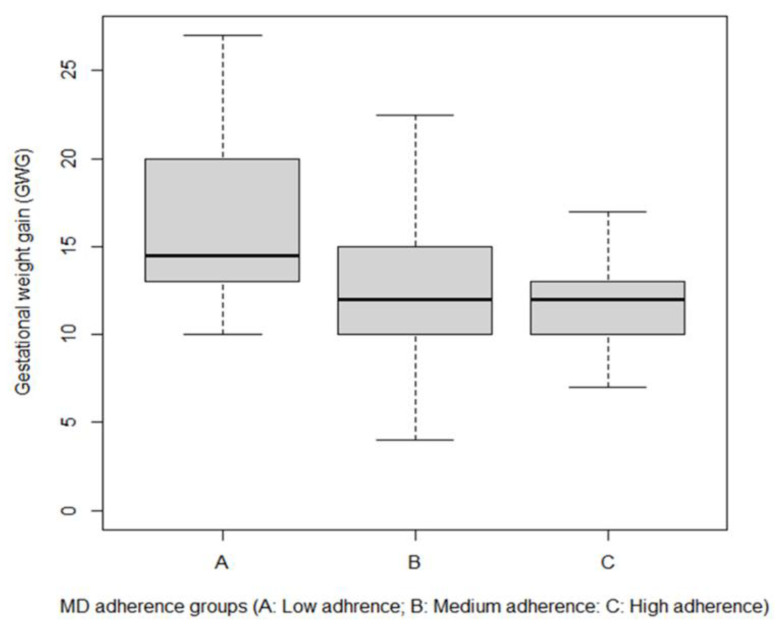
Gestational weight gain in women with low, medium, and high adherence to MD.

The Kruskal–Wallis test demonstrated significant differences between groups and weight gain (*p* < 0.01) and between low and medium-high adherence (Bonferroni post-hoc, *p* < 0.05 and *p* < 0.01, respectively) (Figure 3).

In Table 4, the relationships among the variables are reported. An inverse correlation was found between GWG and MEDAS score pre- and during pregnancy (*p* < 0.001 for both). GWG was also positively correlated with neonatal weight and length (*p* < 0.05 and *p* < 0.001, respectively).

**Figure 4:**
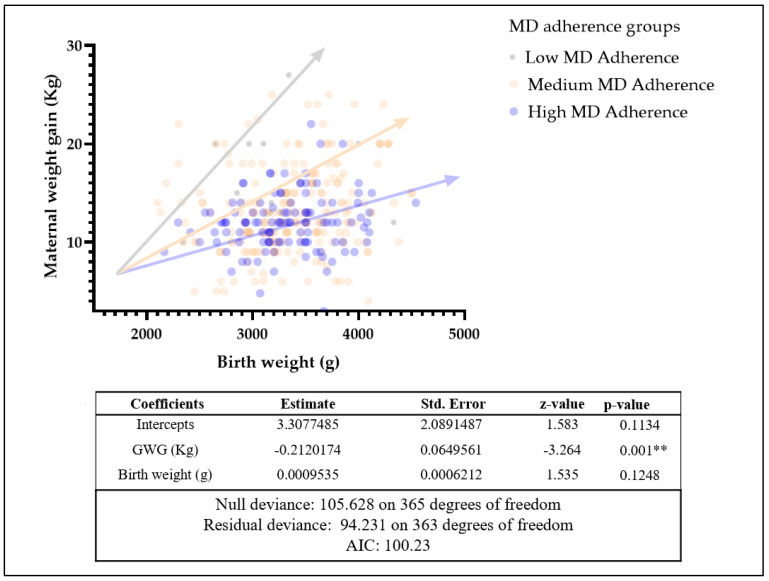
General linear model (GLM) for MD adherence groups on maternal weight gain and birth weight in healthy groups. Statistical significance was attributed as ** *p* < 0.01.

Multiple linear regression with GLM was conducted between GWG and neonatal weight at birth, resulting in significantly different intercepts between different MD adherence groups (*p* < 0.01). The null and residual deviance were analyzed to investigate the possible use for probabilistic characterization, demonstrating this potential employment (Figure 4).

## 4. Discussion

Although the unique nutritional requirements of pregnancy do not allow a generalization with data available for the general population, the relationships found in this study within our data demonstrate a close correlation between weight gain during pregnancy and adherence to MD.

The scientific literature widely supports the importance of lifestyle and dietary habits during pregnancy for the health of mothers and their offspring. It is essential for the health of mothers and their offspring. Maternal nutrition and consumption of a varied and balanced diet from the pre-conception period are necessary to ensure maternal well-being and pregnancy outcomes. Our investigation was developed from the consideration that nutrition is a factor that may affect the development of fetal plasticity during pregnancy and determines the risk of disease in adulthood. The type of diet leads to epigenetic modification in different tissues by altering genetic expression and causing the onset of CDDs [24]. The topic of healthy pregnancies focuses on the maternal diet because it is known that obesity increases the risks for adverse perinatal outcomes, including GDM, large for gestational age newborns, or preeclampsia. Thus, foods rich in vegetables, essential and polyunsaturated fats, and fiber-rich carbohydrates should be promoted, especially in overweight, obese, or diabetic women [25].

The “MeDAP studio”, investigating women’s eating habits in the pre-conception period and during pregnancy, highlighted the critical role of a MD during pregnancy. After classifying our population into the three MD adherence groups, an average adherence was mainly observed in the total sample, with a small percentage of women classified as having low MD adherence. As reported in other studies [26,27,28], it was observed that there is a significant statistical difference in the MEDAS score between the eating habits of women before and during pregnancy, with significantly higher scores than a strong improvement in adherence to MD during pregnancy. Nevertheless, our data disagreed with a similar study that evaluated the prevalence of overweight/obesity dramatically associated with the described trend of low adherence to a MD in its pregnant population [29]. However, in our study sample, the already small percentage of women with low MD adherence had further reduced after pregnancy, thanks to nutritional intervention. Therefore, our data agree with the literature [20], which underlines how pregnant women develop greater adherence to a diet defined as “healthy” throughout pregnancy. In other studies, we found that women also develop greater awareness about the impact of food choices on the fetus’s health [30]. Therefore, these data are also confirmed in this survey, where an improvement in adherence to MD was observed, with a preference for mainly healthier foods than those not suggested by the Mediterranean pattern. In addition, divided by classes of adherence to the MD, there was an adequate consumption of certain typically Mediterranean foods, such as olive oil, vegetables, and fish, before and during pregnancy.

By contrast, for some foods, there was an improvement in consumption, such as the “use of sofrito”, “legumes’’, and “white and red meat”, especially during pregnancy. These dietary changes, especially in the second and third trimesters of pregnancy, are probably related to an increased demand for protein or pregnancy-related iron deficiency. Indeed, these dietary supplies are usually improved during the last weeks of pregnancy, as reported in different studies [31,32,33]. On the contrary, other authors showed a progressive decrease in adherence to the MD and the intake of certain foods such as fruits, vegetables, and fish among the general adult population and pregnant women, especially in the new generations [28,34,35].

In our investigation, we compare obstetrics and neonatal outcomes according to changes in MD adherence during pregnancy. We decided to examine only the uncomplicated pregnancies considering the significant changes in the main obstetric outcomes (possible bias) related to the pregnancy disorders. Mainly, in disagreement with the data of Tomaino et al. [36], our data showed a different birth weight between women with an improved or unchanged MD adherence concerning the women with worsened MD adherence. However, we must underline that the average weight of the newborns in the two groups was normal. No differences were evidenced for the other variables such as gestational age, birth, vaginal delivery, or CS. The only difference related to CS arose from the whole sample, including the complicated pregnancies. These data are probably associated with complicated pregnancies within the entire population, often related to emergency CS.

The GWG has a pivotal role in the considered outcomes in our study. Mediterranean diet interventions may reduce adverse health outcomes, such as cardiovascular events, diabetes, cognitive decline, and other inflammatory-based diseases. A 2021 systematic review by Abdollah et al. reported that higher maternal adherence to a healthy diet was strongly associated with higher birth weights than lower adherence [37].

GWG is influenced by several factors, including maternal comorbidities and psychosocial aspects, especially in countries with low socioeconomic development indicators. In this context, a multi-disciplinary approach (clinical nutritionists, psychologists, and other public health specialists) can help mitigate undesirable outcomes such as a higher GWG [38]. According to the literature [39], our results on GWG are positively correlated with neonatal weight and length.

Knowing that MD has a notable effect on various aspects of human health, we wanted to investigate the potential impact of MD adherence on GWG and different neonatal outcomes. There is evidence that high adherence scores to MD related to early gestational age are associated with varying indices of the body (e.g., weight or length at birth). However, the biological mechanisms have yet to be investigated. According to this evidence in our sample, there is higher birth weight in the offspring of women with improved adherence to MD. Conversely, another study suggested a relationship between a decrease in a woman’s diet quality and increased weight and length at birth [40]. A healthy eating style is associated with a lower risk of premature births and fetal macrosomia [37,41]. This finding may be related to another observation reported in our study. Some women who indicated low adherence to MD were associated with a lower birth weight in relation to their GWG. This result could prove that probably neonatal outcomes are influenced not only by the quantity but also by the quality of the diet during pregnancy.

In agreement with the literature [42], our investigation showed that women who had lower adherence to MD gained more weight than those who had greater adherence to MD. Indeed, we also confirmed that weight gain during pregnancy is directly correlated with the MD adherence degree.

The low adherence group of pregnant women showed a lower birth weight in relation with the same GWG than the medium-high adherence group, confirming our hypothesis for the quality of dietary assessment in association with the GWG as a neonatal outcome key actor.

Even in the last weeks of pregnancy, the medium-high adherence group showed an improved neonatal outcome in relationship with a lower GWG.

Furthermore, by dividing the sample by BMI classes, the average GWG of patients was normal, except for obese women, by the recommendations for GWG [43]. In particular, in our study, the average GWG of women was 9.6 kg, higher than the average GWG recommended by the gynecologist/obstetric guidelines [43], which ranges between 5 and 9.1 kg. As reported in a study by Satpathy et al. [44], pregnancy for pre-obese or obese women must be considered at risk. Therefore, the patient must be informed about the maternal and fetal risks caused by obesity and inappropriate GWG.

Focusing on the central role of the nutritionist in our therapeutic protocol, it is shown that this figure had a strong impact on the group of women followed by a nutritionist compared to women with self-made nutritional advice, but also compared to those followed only by gynecologists. Concerning literature data, our study appears as the first one to investigate the degree of adherence to MD within a research protocol that involves the intervention of several specialists during the gestational period, so we consider it a strength of our findings. Of course, gynecologists and midwives play an essential role in supporting pregnant women and helping them choose a healthy dietary intake. Still, their advice is often general and focused only on food safety issues. This happens due to a lack of time and sufficient resources [45,46]. This approach could lead to new modifiable risk factors appropriate for targeted interventions on important neonatal outcomes, such as birth weight, and may reduce long-term consequences on the child and adult health, such as cardiovascular events, diabetes, and other inflammatory-based diseases. [47,48,49].

All women require nutritional assessment and appropriate, personalized intervention during pre-conception and pregnancy, emphasizing optimizing maternal body mass index and micronutrient reserves [50]. Recommendations during this period could be helpful to ensure optimal maternal and infant health. These aspects could be fundamental also to understanding the importance of the nutrition protocol prescribed by a clinical nutritionist that women should follow during pregnancy, in contrast to leaving them free to eat whatever they want. In this context, the study’s limitations could be the absence of a food diary during the gestational period and the possible survey of the mothers’ eating habits and food consumption after pregnancy.

Nevertheless, among the weak points of our study, we included the homogenous study cohort (i.e., Italian ethnicity). Under the study’s limitations, it is not generalizable to many other populations.

Our study has several limitations. First, it is a retrospective design; second, due to the lack of specific MEDAS questionaries for the obstetrical population, we used a questionnaire validated for the general population. Furthermore, using a priori indices, such as MEDAS, during pregnancies with specific nutritional needs is more prejudicial than in normal condition pregnancies. For example, alcohol consumption is strictly discouraged in pregnancy (even very low consumption as a response to the MEDAS could affect the scoring system, and low consumption of vegetables or high consumption of red and processed meat). This is because these validated indices assign the same value as the score to every item of the questionnaire [51].

## 5. Conclusions

In conclusion, our data demonstrate a close correlation between weight gain during pregnancy and adherence to MD. We also show that most pregnant women improved their MD adherence compared to the pre-pregnancy period. Furthermore, weight gain during pregnancy was significantly influenced by the degree of adherence to MD, emphasizing that the quantity and the quality of food are fundamental aspects of the health of the mother and of the future baby. The diet quality and composition play an important role in determining GWG and its sequelae. Women who follow a nutritional path during pregnancy, accompanied by an expert professional figure, such as a clinical nutritionist, had an improvement in MD adherence. For this, the future goal should be to make the nutritional path in pregnancy more usable in the hospital services and managed by a multidisciplinary team that also includes the nutritionist figure.

## Figures and Tables

**Table 1:** General characteristics of the study population.

Whole Sample (*n* = 501)	
Maternal age	34 [19–53] 34.4 ± 4.86
<35 years	273 (54.5)
≥35 years	228 (45.5)
Pre-pregnancy weight (kg)	58.5 [41–129] 61.0 ± 11.4
Height (cm)	165.0 [148–170] 165.4 ± 0.34
Pre-pregnancy body mass index BMI (kg/cm^2^)	21.48 [16.0–50.4] 22.4 ± 4.1
Underweight (≤18.4 kg/cm^2^)	36 (7.19)
Normal weight (18.5–24.9 kg/cm^2^)	376 (75.05)
Overweight (25.0–29.9 kg/cm^2^)	65 (12.97)
Obesity (≥30 kg/cm^2^)	24 (4.79)
Gestational weight gain (kg)	12.0 [0–31] 12.6 ± 4.5
Nulliparous	287 (57.3)
Italian	476 (95.01)
Not-Italian	25 (4.99)
Primary School	5 (1.0)
College	147 (29.34)
Bachelor degree	274 (54.69)
Ph.D.	75 (14.97)
Pregnancy complications	
COVID-19	15 (11.1)
Diabetes	48 (35.6)
Cholestasis	11 (8.1)
Hypertension and preeclampsia	19 (14.1)
Others	42 (31.1)

Values are expressed as median and IQR (M [IQR]) and mean ± SD for continuous variables or as number and percentage (*n* (%)) for categorical variables.

**Table 2:** Newborn characteristics.

	Whole Sample (*n* = 501)
Male	269 (53.7)
Female	232 (46.3)
Gestational age birth (weeks)	39.2 [2.1]
Vaginal delivery	368 (73.5)
Cesarean section	133 (26.5)
Birth weight <10th percentile (SGA)	44 (8.8)
Birth weight between 10 and 90 percentile (AGA)	385 (76.8)
Birth weight >90th percentile (LGA)	72 (14.4)
Birth weight <2500 g	29 (5.8)
Birth weight 2500–4000 g	469 (93.6)
Birth weight ≥4500 g	3 (0.6)
Birth length	50.3 [3.2]
Admission to NICU	27 (5.4)
Admission to SCBU	11 (2.2)

Values are expressed as median and IQR (M [IQR]) for continuous variables or as number and percentage (*n* (%)) for categorical variables.

**Table 3:** Correlations between MD adherence and neonatal and obstetrics outcomes.

	Improved or Unchanged Adherence (*n* = 290)	Worsened Adherence (*n* = 76)	*p*
Birth weight (g)	3363.8 [458.3]	3220.1 [502.0]	0.02 *
Birth length (cm)	50.4 [2.3]	50.2 [3.3]	0.42 †
Gestational age birth (weeks)	39.5 [1.2]	39.3 [1.7]	0.52 †
Vaginal delivery	231 (79.7)	55 (72.4)	0.17 χ^2^
Cesarean section	59 (20.3)	21 (27.6)	

Differences between improved or unchanged and worsened groups were compared by *t*-test and Wilcoxon test (†). Statistical significance was attributed as * *p* < 0.05; * *p* < 0.05 for linearity (Pearson χ^2^ test). The Shapiro–Wilk test was performed to evaluate the variable distribution. Variables are considered non-normally distributed for *p* < 0.05.

**Table 4:** Correlation between neonatal and obstetric outcomes and MEDAS in uncomplicated women (*n* = 366).

	BMI	Weight Gain	Gestational Age Birth	Birth Weight	Birth Length	SCORE before Pregnancy	SCORE during Pregnancy
Age	0.67	(−0.15) **	(−0.14) **	(−0.19)	(−0.04)	0.05	(−0.16)
	0.20	0.004	0.008	0.72	0.44	0.37	0.76
BMI		(−0.12) *	(−0.01)	0.08	0.02	(−0.04)	(−0.02)
		0.02	0.83	0.11	0.76	0.41	0.74
weight gain			0.13 *	0.14 **	0.18 **	(−0.18) **	(−0.17) **
			0.01	0.01	<0.001	<0.001	<0.001
Gestational age birth				0.34 **	0.34 **	(−0.05)	(−0.03)
				<0.001	<0.001	0.34	0.53
Birth weight					0.54 **	(−0.11) *	(−0.03)
					<0.001	0.03	0.59
Birth length						(−0.07)	(−0.07)
						0.17	0.21
SCORE before pregnancy							0.74 **
							<0.001

Data are presented as Pearson’s correlation coefficients and *p* values. Statistical significance was attributed as * *p* < 0.05; ** *p* < 0.01.

## Data Availability

All data generated or analyzed during this study are included in this published article.

## Supplementary Materials

The following supporting information can be downloaded at: https://www.mdpi.com/article/10.3390/ijerph19148497/s1, File S1: questionnaires.
