# Supplementary material for: Adherence to Mediterranean Diet and Its Association with Maternal and Newborn Outcomes
Source: Int J Environ Res Public Health. 2022 Jul 12;19(14):8497. doi: 10.3390/ijerph19148497 (PMC9321919; doi:10.3390/ijerph19148497)
Supplement: Supplementary file 1 [file ijerph-19-08497-s001.zip › ijerph-1740779-supplementary/ijerph-1740779-File S1. Questionnaire.pdf]

Questionnaire

1. Age

2. Nationality

3. Educational qualification

- Primary School
- Secondary school
- Degree
- Master's or PhD

4. Write your height (cm)

5. Write your weight before pregnancy (kg)

6. How many kilos did you gain in the last pregnancy?

7. On average how many kilos did you gain in previous pregnancies?

____

8. Have you had any other pregnancies?

- ◯ Yes
- ◯ No

9. How many spontaneous births?

- ◯ 0
- ◯ 1
- ◯ 2
- ◯ > 3

10. How many spontaneous miscarriages?

- ◯ 0
- ◯ 1
- ◯ 2
- ◯ > 3

11. How many IVGs?

- ◯ 0
- ◯ 1
- ◯ 2
- ◯ > 3

12. Do you suffer from any major diseases?

- Diabetes
- Thyroidopathies
- Hypertension
- Polycystic ovary
- Heart diseases
- Genetic diseases
- Epilepsy
- None
- Other: ____________________

13. Do you usually take any drugs?

- Yes
- No

14. If the answer is "yes" write which drugs, otherwise answer "no".

____

____

____

____

____

15. What type of birth did you undergo?

- Natural
- Cesarean

16. At what week of gestation did you give birth?

____

17. How much did the baby weigh at birth (gr)?

____

18. How long was the baby at birth (cm)?

____

19. Where was the baby admitted after birth?

- Nest (cradle near the mother)
- Neonatal Intensive Care
- Other: ____

20. If the answer is "Neonatal Intensive Care", write the reason

____

21. Did you develop pathologies during pregnancy?

- No
- Gestational diabetes
- Gestational hypertension
- Preeclampsia
- Eclampsia
- Cholestasis
- Toxoplasmosis
- Cytomegalovirus
- Chickenpox
- Covid-19
- Other: ______________________

22. If you answered "Covid 19" to the previous question, specify your gestation period

____

23. Were you often on a diet before pregnancy?

- Yes
- No

24. Were you worried about your weight before pregnancy?

- Yes
- No

25. Were you on a diet during pregnancy?

- Yes
- No

26. If the answer is yes, who was it followed by?

- Nutritionist
- Gynecologist
- Family Doctor
- Other: ______________________________

27. Were you worried about your weight during pregnancy?

- Yes
- No

28. Did you attend a birth accompaniment course during your last pregnancy?

- Yes
- No

29. If so, has the topic of nutrition and supplements in pregnancy been discussed during the course?

- Yes
- No

30. Did you use any food or vitamin supplements when you were pregnant?

- Yes
- No

31. If the answer is "yes", specify which ones

____

32. Are you vegetarian?

- Yes
- No

33. Are you vegan?

- Yes
- No

34.Has your diet changed during pregnancy or was it your usual one?

- No
- Yes, I have decreased the amount of portions
- Yes, I have increased the portion size
- Yes, I have eliminated some foods

35. If you have reduced quantities, at what time of pregnancy?

- Never
- In the first trimester
- In the second trimester
- In the third trimester

36. If you have increased quantities, at what time of pregnancy?

- Never
- In the first trimester
- In the second trimester
- In the third trimester

37. Have you avoided some foods for pathologies or intolerances?

- No
- Yes

38. If the answer is "yes", specify the reason

____

39. If the answer is "yes", specify which foods

40. Have you introduced new products and food during pregnancy?

- No
- Yes

41. If the answer is "yes", specify which ones

42. If the answer is "yes”, specify the trimester in which you have introduced new foods.

- No, I haven’t introduced any new foods
- yes, from the 1°
- yes, from the 2°
- yes, from the 3°

43. Did you smoke during pregnancy?

- Yes
- No

44. Did you drink alcohol during pregnancy?

- Yes
- No

45. Did you take narcotic substances during pregnancy?

- ◯ Tranquilizer
- ◯ Cannabis
- ◯ Hashish
- ◯ Nothing
- ◯ Other: ____________________

Please indicate with an X the answer you most identify with (it can also indicate more than one answer).

46. How many times do you/did you practise pysical exercise?

	Never	1-2 times a week	3-4 times a week	> 5 times a week
Before the pregnancy	☐	☐	☐	☐
During pregnancy	☐	☐	☐	☐

This part of the questionnaire contains a series of statements regarding the lifestyle or eating habits followed before pregnancy.

47. BEFORE PREGNANCY did you use olive oil as your main cooking fat?

- Yes
- No

48. BEFORE PREGNANCY Did you use 4 or more tablespoons of olive oil per day (including oil for frying, salad dressing, meals outside the home, etc.)?

- Yes
- No

49. BEFORE PREGNANCY Did you eat 2 or more servings of vegetables per day (1 serving = 200g)?

- Yes
- No

50. BEFORE PREGNANCY Did you consume 3 or more servings of fruit per day (including juices or natural centrifuges)?

- Yes
- No

51. BEFORE PREGNANCY Did you eat less than 1 serving (= 100-150g) of red meat, hamburgers or meat products (sausages etc) every day?

- Yes
- No

52. BEFORE PREGNANCY Did you consume less than 1 serving (12 g) of butter, margarine or cream per day?

- Yes
- No

53. BEFORE PREGNANCY Did you drink less than 1 glass (= 100ml) of carbonated sugary drinks per day?

- Yes
- No

54. BEFORE PREGNANCY Did you drink 7 or more glasses (= 100 ml) of red wine a week?

- Yes
- No

55. BEFORE PREGNANCY did you eat 3 or more portions (1 portion = 150g boiled) of legum a week?

- Yes
- No

56. BEFORE PREGNANCY did you eat 3 or more portions (1 portion = 150 g) of fish or sea fruit a week?

- Yes
- No

57. BEFORE PREGNANCY did you consume less than 3 times a week of sweets or commercial pastries (no homemade) as biscuits or cakes?

- Yes
- No

58. BEFORE PREGNANCY Did you consume 3 or more servings (1 serving = 30g) of dry fruit (walnuts, almonds, hazelnuts, etc.) a week?

- Yes
- No

59. BEFORE PREGNANCY Did you prefer white meat (chicken, turkey, rabbit) instead of red meat (veal, pork, hamburger, sausage)?

- Yes
- No

60. BEFORE PREGNANCY Did you use the "mixture" (tomato, onion, garlic or leek cooked with olive oil) to cook vegetables, pasta, rice or other dishes 2 or more times a week?

- Yes
- No

This part of the questionnaire contains a series of statements regarding the lifestyle, or eating habits, followed during the pregnancy period.

61. DURING PREGNANCY Did you use olive oil as your main cooking fat?

- Yes
- No

62. DURING PREGNANCY Did you use 4 or more tablespoons of olive oil per day (including oil for frying, salad dressing, meals outside the home, etc.)?

- Yes
- No

63. DURING PREGNANCY Did you eat 2 or more servings of vegetables per day (1 serving = 200g)?

- Yes
- No

64. DURING PREGNANCY Did you consume 3 or more servings of fruit per day (including natural juices)?

- Yes
- No

65. DURING PREGNANCY Did you consume less than 1 serving (= 100-150g) of red meat, hamburgers or meat products (sausages etc) every day?

- Yes
- No

66. DURING PREGNANCY Did you consume less than 1 serving (12 g) of butter, margarine or cream per day?

- Yes
- No

67. DURING PREGNANCY Did you drink less than 1 glass (= 100 ml) of sugary or carbonated drinks per day?

- Yes
- No

68. DURING PREGNANCY Did you drink 7 or more glasses (= 100 ml) of red wine a week?

- Yes
- No

69. DURING PREGNANCY did you consume 3 or more portions (1 portion = 150g boiled) of legumes a week?

- Yes
- No

70. DURING PREGNANCY Did you eat 3 or more portions (1 portion = 150 g) of fish or sea fruit a week?

- Yes
- No

71. DURING PREGNANCY did you consume less than 3 times a week sweets or commercial pastries (not homemade)?

- Yes
- No

72. DURING PREGNANCY Did you consume 3 or more servings (1 serving = 30g) of dry fruit (walnuts, almonds, hazelnuts, etc.) a week?

- Yes
- No

73. DURING PREGNANCY Did you prefer white meat (chicken, turkey, rabbit) or red meat (veal, pork, hamburger, sausage)?

- Yes
- No

74. DURING PREGNANCY Did you use the "mixture" (tomato, onion, garlic or leek cooked with olive oil) to cook vegetables, pasta, rice or other dishes 2 or more times a week?

- Yes
- No

Kindly, for each row (before pregnancy, first, second and third trimester) indicate with an X the answer with which you most identify.

75. How often have you eaten WHITE MEAT?

Select all applicable items.

	Daily	Very Often	1-2 times a week	Never
Before pregnancy	☐	☐	☐	☐
First	☐	☐	☐	☐
Second	☐	☐	☐	☐
Third	☐	☐	☐	☐

76. How often have you eaten RED MEAT?

Select all applicable items.

	Daily	Very Often	1-2 times a week	Never
Before pregnancy	☐	☐	☐	☐
First	☐	☐	☐	☐
Second	☐	☐	☐	☐
Third	☐	☐	☐	☐

77. How often have you eaten FISH?

Select all applicable items.

	Daily	Very Often	1-2 times a week	Never
Before pregnancy	☐	☐	☐	☐
First	☐	☐	☐	☐
Second	☐	☐	☐	☐
Third	☐	☐	☐	☐

78. How often have you eaten EGGS?

Select all applicable items.

	Daily	Very Often	1-2 times a week	Never
Before pregnancy	☐	☐	☐	☐
First	☐	☐	☐	☐
Second	☐	☐	☐	☐
Third	☐	☐	☐	☐

79. How often have you eaten LEGUMES?

Select all applicable items.

	Daily	Very Often	1-2 times a week	Never
Before pregnancy	☐	☐	☐	☐
First	☐	☐	☐	☐
Second	☐	☐	☐	☐
Third	☐	☐	☐	☐
